# Metabolic Enzyme Alterations and Astrocyte Dysfunction in a Murine Model of Alexander Disease With Severe Reactive Gliosis

**DOI:** 10.1016/j.mcpro.2021.100180

**Published:** 2021-11-20

**Authors:** Michael R. Heaven, Anthony W. Herren, Daniel L. Flint, Natasha L. Pacheco, Jiangtao Li, Alice Tang, Fatima Khan, James E. Goldman, Brett S. Phinney, Michelle L. Olsen

**Affiliations:** 1 Vulcan Biosciences, Birmingham, Alabama, USA; 2 University of California at Davis Proteomics Core, Davis, California, USA; 3 Luxumbra Strategic Research, Arlington, Virginia, USA; 4 Department of Cell, Developmental, and Integrative Biology, University of Alabama at Birmingham, Birmingham, Alabama, USA; 5 Graduate Program in Genetics, Bioinformatics, and Computational Biology, Virginia Tech, Blacksburg, Virginia, USA; 6 School of Neuroscience, Virginia Tech, Blacksburg, Virginia, USA; 7 Department of Pathology and Cell Biology, Columbia University Medical Center, New York, New York, USA

**Keywords:** Alexander disease, reactive gliosis, astrocytes, Ugt8, Fabp7, μDIA, micro-data-independent acquisition, Acadl, Long-chain specific acyl-CoA dehydrogenase, mitochondrial, Acot1, Acyl-coenzyme A thioesterase 1, Acot2, Acyl-coenzyme A thioesterase 2, Acox1, peroxisomal acyl-coenzyme A oxidase 1, Acox3, Peroxisomal acyl-coenzyme A oxidase 3, Acsl3, long-chain-fatty-acid--CoA ligase 3, Acsl6, long-chain-fatty-acid--CoA ligase 6, Aldh1l1, Cytosolic 10-formyltetrahydrofolate dehydrogenase, Apoa1, apolipoprotein A-I, Atp1b2, sodium/potassium-transporting ATPase subunit beta-2, AxD, Alexander disease, BCA, bicinchoninic acid assay, Bsn, protein bassoon, C1qa, complement C1q subcomponent subunit A, C1qb, complement C1q subcomponent subunit B, C1qc, complement C1q subcomponent subunit C, Ca2, carbonic anhydrase 2, Cat, catalase, Ccnd2, G1/S-specific cyclin-D2, Cd44, Cd44 antigen, Cnp, 2′,3′-cyclic-nucleotide 3′-phosphodiesterase, CNS, central nervous system, Cryab, alpha-crystallin B chain, Cryl1, lambda-crystallin homolog, Ctsd, cathepsin D, DAVID, database for annotation, visualization and integrated discovery, Ddx3x, ATP-dependent RNA helicase DDX3X, Dlg4, disks large homolog 4, Ephx1, epoxide hydrolase 1, Fabp7, fatty acid binding protein, brain, Fasn, fatty acid synthase, GABA, gamma aminobutyric acid, Gan, gigaxonin, GAPDH, glyceraldehyde 3 phosphate dehydrogenase, Gclc, glutamate--cysteine ligase catalytic subunit, Gclm, glutamate--cysteine ligase regulatory subunit, GFAP, glial fibrillary acidic protein, Gja1, gap junction alpha-1 protein, Gpr37l1, prosaposin receptor GPR37L1, Gpx1, glutathione peroxidase 1, Gpx3, glutathione peroxidase 3, Gsr, glutathione reductase, mitochondrial, Gss, glutathione synthetase, Gsta3, glutathione S-transferase A3, Gstm1, glutathione S-transferase Mu 1, Gsto1, glutathione S-transferase omega-1, Hmgcs2, hydroxymethylglutaryl-CoA synthase, mitochondrial, Hsp27, heat shock protein beta-1, Idh2, isocitrate dehydrogenase [NADP], mitochondrial, Ilk, integrin-linked protein kinase, Kcnj10, ATP-sensitive inward rectifier potassium channel 10, KEGG, Kyoto encyclopedia of genes and genomes, LC3, microtubule-associated protein 1A/1B-light chain 3, LPA, lysophosphatidic acid, Mag, myelin-associated glycoprotein, Mbp, myelin basic protein, MDD, major depressive disorder, Mlc1, membrane protein MLC1, Mobp, myelin-associated oligodendrocyte basic protein, Mog, myelin-oligodendrocyte glycoprotein, MS2, MS/MS or tandem mass spectrometry, Nadk2, NAD kinase 2, mitochondrial, Ndrg2, protein NDRG2, Nfe2l2, nuclear factor erythroid 2-related factor 2, Ntrk2, BDNF/NT-3 growth factors receptor, Pck2, phosphoenolpyruvate carboxykinase [GTP], mitochondrial, Pgd, 6-phosphogluconate dehydrogenase, decarboxylating, Plp1, myelin proteolipid protein, Plpp3, phospholipid phosphatase 3, PPAR, peroxisome proliferator-activated receptor, PPP, pentose phosphate pathway, Prdx1, peroxiredoxin-1, Prdx6, peroxiredoxin-6, PRM-MS, parallel reaction monitoring-mass spectrometry, Pygb, glycogen phosphorylase, brain form, Rack1, receptor of activated protein C kinase 1, RFs, Rosenthal fibers, Rps27a, ubiquitin-40S ribosomal protein S27a, Sfxn5, sideroflexin-5, Slc1a2, excitatory amino acid transporter 2, Slc4a4, electrogenic sodium bicarbonate cotransporter 1, Slc6a11, sodium- and chloride-dependent GABA transporter 3, Slc6a17, sodium-dependent neutral amino acid transporter SLC6A17, Slc16a1, monocarboxylate transporter 1, Slc25a18, mitochondrial glutamate carrier 2, Slc27a1, long-chain fatty acid transport protein 1, Slc38a3, sodium-coupled neutral amino acid transporter 3, S1pr1, sphingosine 1-phosphate receptor 1, Snap25, synaptosomal-associated protein 25, Sorbs1, Sorbin and SH3 domain-containing protein 1, Sparcl1, SPARC-like protein 1, Sqstm1, sequestosome-1, SRM, selected reaction monitoring, Stat3, signal transducer and activator of transcription 3, Stxbp5l, syntaxin-binding protein 5-like, TBI, traumatic brain injury, TFA, trifluoro acetic acid, TIC, total ion current, Ttyh1, protein tweety homolog 1, Txnrd1, thioredoxin reductase 1, cytoplasmic, Ugp2, UTP--glucose-1-phosphate uridylyl transferase, Ugt8, 2-hydroxyacylsphingosine 1-beta-galactosyltransferase, Vim, vimentin

## Abstract

Alexander disease (AxD) is a rare and fatal neurodegenerative disorder caused by mutations in the gene encoding glial fibrillary acidic protein (GFAP). In this report, a mouse model of AxD (*GFAP*^*Tg*^;*Gfap*^+/*R236H*^) was analyzed that contains a heterozygous R236H point mutation in murine *Gfap* as well as a transgene with a *GFAP* promoter to overexpress human GFAP. Using label-free quantitative proteomic comparisons of brain tissue from *GFAP*^*Tg*^;*Gfap*^+/*R236H*^ *versus* wild-type mice confirmed upregulation of the glutathione metabolism pathway and indicated proteins were elevated in the peroxisome proliferator-activated receptor (PPAR) signaling pathway, which had not been reported previously in AxD. Relative protein-level differences were confirmed by a targeted proteomics assay, including proteins related to astrocytes and oligodendrocytes. Of particular interest was the decreased level of the oligodendrocyte protein, 2-hydroxyacylsphingosine 1-beta-galactosyltransferase (Ugt8), since *Ugt8*-deficient mice exhibit a phenotype similar to *GFAP*^*Tg*^;*Gfap*^+/*R236H*^ mice (*e.g.*, tremors, ataxia, hind-limb paralysis). In addition, decreased levels of myelin-associated proteins were found in the *GFAP*^*Tg*^;*Gfap*^+/*R236H*^ mice, consistent with the role of Ugt8 in myelin synthesis. Fabp7 upregulation in *GFAP*^*Tg*^;*Gfap*^+/*R236H*^ mice was also selected for further investigation due to its uncharacterized association to AxD, critical function in astrocyte proliferation, and functional ability to inhibit the anti-inflammatory PPAR signaling pathway in models of amyotrophic lateral sclerosis (ALS). Within Gfap^+^ astrocytes, Fabp7 was markedly increased in the hippocampus, a brain region subjected to extensive pathology and chronic reactive gliosis in *GFAP*^*Tg*^;*Gfap*^+/*R236H*^ mice. Last, to determine whether the findings in *GFAP*^*Tg*^;*Gfap*^+/*R236H*^ mice are present in the human condition, AxD patient and control samples were analyzed by Western blot, which indicated that Type I AxD patients have a significant fourfold upregulation of FABP7. However, immunohistochemistry analysis showed that UGT8 accumulates in AxD patient subpial brain regions where abundant amounts of Rosenthal fibers are located, which was not observed in the *GFAP*^*Tg*^;*Gfap*^+/*R236H*^ mice.

## Highlights

• Models of AxD provide insight into functions of astrocytes related to neuropathology.
• PPAR pathway found upregulated in AxD mice may be neuroprotective. Fatty acid binding protein (Fabp7) was upregulated in AxD mice and human CNS samples.
• Depleted levels in the classical constituents of myelin were observed in AxD mice.
• UGT8 may be related to myelin deficits in AxD.

## Graphical abstract

**Figure:**
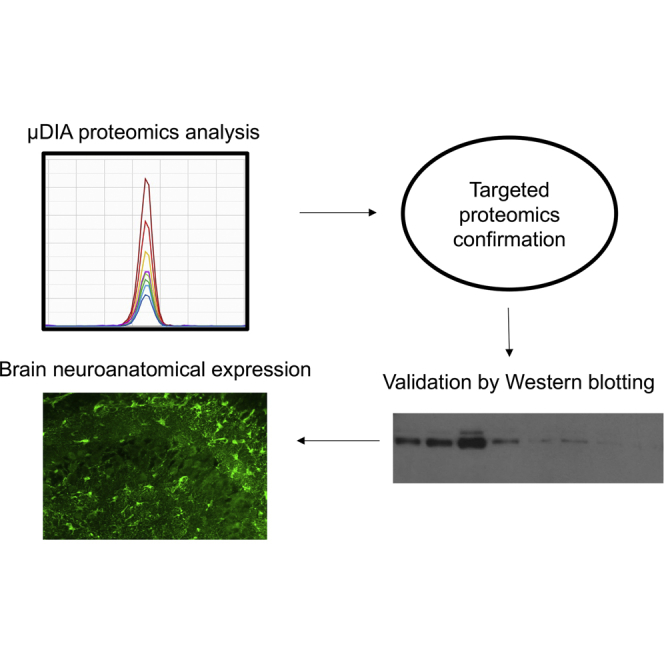

## In Brief

The article contains the first whole brain proteomic survey from a mouse model of Alexander disease (AxD). Several novel findings include activation of the PPAR signaling pathway, which has been reported to be protective in models of amyotrophic lateral sclerosis (ALS). Another finding related to the gliosis phenotype in AxD mice was the upregulation of fatty acid binding protein 7 (FABP7), which induces an NF-κB inflammatory response and counteracts the anti-inflammatory effects of PPAR signaling.

Astrocytes are a major population of cells in the mammalian central nervous system (CNS) (1). Although once regarded as merely passive support cells, they are now recognized to play critical functional roles in both health and disease. During development, astrocytes contribute to the formation and refinement of neural circuits by regulating synapse formation, function, and pruning (2). In the mature CNS, astrocytes play an active role in energy metabolism (3), extracellular potassium homeostasis (4), and the recycling of neurotransmitters such as glutamate (5) and GABA (6). In response to CNS insults, astrocytes undergo a process of “reactive gliosis” characterized by cellular hypertrophy, upregulation of the glial fibrillary acidic protein (GFAP), and altered gene expression. In severe cases, these changes are accompanied by mild astrocyte proliferation and permanent scarring of the tissue (7). Astrocytes have been implicated in the pathophysiology of a wide spectrum of neurological disorders and injuries, including autism (8), epilepsy (9), amyotrophic lateral sclerosis (ALS) (10), major depressive disorder (MDD) (11), traumatic brain injury (TBI) (12), and Alexander disease (AxD) (13).

AxD is unique among other diseases because the primary disorder originates in astrocytes due to gain-of-function mutations in the human *GFAP* gene (13), which is expressed predominantly in astrocytes, thereby isolating how these cells contribute to neuropathology. Accordingly, AxD has been classified as an astrogliopathy—that is, a primary disease of astrocytes (13). Clinically, AxD is often fatal, with early-onset (Type I) cases manifesting with seizures and macrocephaly, whereas late-onset (Type II) cases are associated with autonomic dysfunction and motor disturbances (14). The neuropathology of AxD is characterized by pronounced reactive gliosis, the presence of cytoplasmic protein aggregates known as Rosenthal fibers (RFs), and varying degrees of neurodegeneration and white matter deficits (13). At the molecular level, AxD brain tissues have reduced levels of the astrocytic glutamate transporter (15, 16), as well as classical signs of neurodegeneration, including the induction of markers of autophagy (*e.g.*, LC3) (17) and oxidative stress (18).

Based on quantitative gene expression analyses, prior mouse model studies have shown that AxD is characterized by mRNA changes suggestive of CNS inflammation with accompanying microglial activation (19), as well as the induction of glutathione antioxidant response genes (20). However, the mRNA levels quantified in these studies may not correlate with actual protein concentrations (21). Additionally, one of these prior reports used a mouse model lacking an overt phenotype (20), and the other only interrogated the hippocampus (19). To extend these previous reports to the protein level in whole brain, LC-MS/MS-based proteomics was performed on a severe mouse model of AxD (referred to hereafter as *GFAP*^*Tg*^;*Gfap*^+/*R236H*^), which harbors a disease-causing heterozygous point mutation at R236H in murine *Gfap* as well as elevated levels of GFAP due to a human *GFAP* transgene expressed in astrocytes (18). These mice develop motor abnormalities (front limb weakness) and die at approximately postnatal day 35 from convulsive seizures, thus recapitulating some of the clinical features of AxD (19). Moreover, *GFAP*^*Tg*^;*Gfap*^+/*R236H*^ mice display AxD-like neuropathological and molecular changes that include pronounced reactive gliosis, the presence of RFs (22), an inflammatory response characterized by microglial activation, and reduced levels of the astrocytic glutamate transporter (23).

## Experimental Procedures

### Tissue Procurement and Homogenization

All mouse protocols used in the study were approved by the University of Alabama at Birmingham Institutional Animal Care and Use Committee. Whole brains, including brain stem and olfactory bulbs, were obtained from 25-day-old female *GFAP*^*Tg*^;*Gfap*^+/*R236H*^ mice (18) and wild-type littermate controls on a FVB/N background. Brains were harvested by rapid decapitation, frozen on dry ice, and stored at −80 °C. The brain samples were Dounce homogenized in ice-cold Milli-Q water (EMD Millipore). Half of the resulting homogenate volume obtained from each sample was immediately placed in a urea buffer to reach a final concentration of 8 M urea/100 mM Tris-HCl pH 8.3 and stored at −80 °C until used for mass spectrometry sample preparation. The other half of the resulting homogenate from each brain was used for Western blotting and immediately mixed with SDS to obtain a final concentration of 5% (w/v) SDS, followed by heating for 30 min at 99 °C. Protein concentrations in the urea and SDS buffers were determined using the bicinchoninic acid (BCA) assay (Pierce).

The use of all human tissues in the study adheres to the Declaration of Helsinki principles. Frozen human brain tissue for Western blotting was Dounce homogenized in RIPA lysis buffer consisting of 50 mM Tris, 150 mM Nacl, 1% Triton X-100 (v/v), 0.5% sodium deoxycholate (w/v), 0.1% SDS (w/v) with the manufacturer’s recommended protocol for the protease inhibitor (Sigma-Aldrich, catalog # P8340) and phosphatase inhibitor (Sigma-Aldrich catalog # P0044).

### Immunohistochemistry

*GFAP*^*Tg*^;*Gfap*^+/*R236H*^ mice and wild-type littermate controls were anesthetized with a peritoneal injection of ketamine (100 mg/kg) and perfused with 4% (w/v) paraformaldehyde between postnatal days 21 to 25. Tissue sections (100 μm) were made using a Vibratome (Oxford Instruments) and incubated in blocking buffer (BB, 10% serum, 3% (v/v) Triton X-100 in PBS) for 60 min at room temperature. The tissue slices were then incubated in primary antibody in diluted BB (1:3) overnight at 4 °C with gentle rocking (Ugt8, Proteintech, catalog #17982-1, dilution factor 1:50 and Fabp7, Abcam, catalog #ab32423, dilution factor 1:100, Gfap, EMD Millipore catalog #MAB360, dilution factor 1:1000). Next, the slices were washed three times in diluted BB (15 min each) and incubated in a goat anti-rabbit Alexa-Fluor 488 secondary antibody (Thermo Scientific, catalog #A11008, dilution factor 1:500) for 1 h at room temperature. Following secondary antibody incubation, the slices were washed three times in diluted BB for 15 min, once in PBS, and then mounted onto glass coverslips. Fluorescent images were acquired with an Axio Observer D1 microscope (Zeiss).

Immunostaining of human brain specimens was performed on isocortex and subcortical white matter from autopsied patients and controls. Tissues were fixed in 10% formalin, embedded in paraffin, and processed for immunohistochemistry. Primary antibodies against FABP7 (rabbit monoclonal Abcam catalog # ab92455, diluted 1:500) and UGT8 (Proteintech catalog # 17982-1 diluted 1:250) were immunostained after 20 min of antigen retrieval by boiling in citrate buffer. Stains were visualized with an ABC immunoperoxidase kit (Vector Labs), and the slides were counterstained with hematoxylin and examined with an Olympus BX40 microscope.

### Western Blotting

For the mouse Ugt8 blot, 10 μg of total protein from each sample in 5% (w/v) SDS was analyzed. The samples were boiled in Laemmli sample buffer for 15 min at 60 °C and electrophoresed with a 4 to 20% gradient precast mini-PROTEAN TGX gel (Bio-rad) for 1 h at 200 V. Proteins were transferred to an Immobilon-P PVDF membrane (EMD Millipore) for 1 h at room temperature at 100 V, followed by blocking with 10% (w/v) nonfat dry milk/0.1% (v/v) Tween-20/TBS (diluent buffer) for 1 h at room temperature. The blot was then incubated with a chicken anti-GAPDH primary antibody (EMD Millipore, dilution factor 1:2000) as a loading control for 1 h at room temperature. Next, the blot was washed three times in 10% diluent buffer, incubated for 1 h at room temperature with a goat anti-chicken secondary antibody (Santa Cruz Biotechnology, dilution factor 1:2000), and washed three times with diluent buffer. The blot was developed with Classico chemiluminescent reagent (EMD Millipore) using an autoradiography film developing system (Denville Scientific). Blots were probed with primary antibody rabbit anti-Ugt8 (Proteintech, dilution factor 1:500) similar to GAPDH with the following modifications: the primary antibody incubation was performed overnight at 4 °C, and a goat anti-rabbit secondary antibody (Santa Cruz Biotechnology, dilution factor 1:2000) was used. Proteins were quantified using Image Studio Lite (Li-Cor Biosciences) and Origin2015 (OriginLab) with relative Ugt8 intensities normalized to GAPDH followed by Log_2_ transforming the ratios and applying an unpaired *t* test statistical analysis.

For the human brain tissue Western blots, 10 μg of total protein from each sample was heated to 65 °C for 10 min in 2× sample buffer (100 mM Tris, 10% SDS (w/v), 2% Bromophenol blue (w/v), 1M DTT) and electrophoresed with a 4 to 20% gradient precast mini-PROTEAN TGX gel (Bio-Rad) for 30 min at 200 V. Proteins were transferred to a Transblot Turbo Mini-size Nitrocellulose Membrane (Bio-Rad) for 3 min using the Trans-blot Turboblot Transfer system (Bio-Rad), followed by blocking for 1 h with Odyssey Blocking Buffer (Li-COR Biosciences) diluted 1:1 with TBS at room temperature. The blots were then incubated with either rabbit UGT8 (Proteintech catalog # 17982-1-AP), rabbit FABP7 (Proteintech catalog # 51101-1-AP) antibody at 1:1000, or rabbit Dako GFAP (Agilent, catalog #Z0334) antibody at 1:10,000, and mouse anti-actin primary antibody (Millipore catalog # A5441) at 1:10,000 overnight at 4 °C. Next, the blot was washed three times in TBST for 5 min each prior to incubation for 1 h at room temperature with goat anti-rabbit and goat anti-mouse secondary antibody (Li-COR Biosciences) at 1:10,000. Blots were washed again three times for 5 min in TBST, then washed once for 5 min in TBS prior to imaging using a Li-COR imaging system. Proteins were quantified using Image Studio Lite (Li-COR Biosciences) and Prism 9.1.0 (GraphPad) with relative UGT8 and FABP7 intensities normalized to actin followed by applying an unpaired *t* test statistical analysis.

### In-solution Trypsin Digestion for Untargeted Proteomics

An amount corresponding to 150 μg of protein from each sample was processed using an S-Trap mini column (Protifi) according to the manufacturer’s instructions. Briefly, samples were supplemented to a final buffer composition of 5% (w/v) SDS/50 mM ammonium bicarbonate, reduced in 20 mM DTT for 20 min at 37 °C, and alkylated in 40 mM iodoacetamide for 30 min at room temperature. Trypsin was added at a (1:25) ratio (enzyme:total protein), digested for 1 h at 47 °C, then spiked again with the same amount of trypsin, and digested for another hour at 47 °C. Eluted peptides were dried by vacuum centrifugation, reconstituted in 2% acetonitrile/0.1% TFA, and peptide concentrations were determined by fluorometric peptide assay (Pierce) prior to injection.

### Untargeted Proteomics LC-MS/MS Acquisition

A microgram of peptides from each sample was analyzed by LC-MS/MS on an Orbitrap Fusion Lumos mass spectrometer (Themo Scientific) in conjunction with an UltiMate 3000 RSLCnano UHPLC and an EASY-Spray nanoelectrospray ionization source operating in positive ion mode. Peptides were loaded on an Acclaim PepMap 100 75 μm × 20 mm 3 μm C18 reverse-phase trap before being separated using an EASY-Spray 75 μm × 250 mm 2 μm C18 reverse-phase analytical column (Thermo Scientific). Peptides were eluted with an increasing percentage of acetonitrile over a 120 min gradient with a flow rate of 200 nl/min at 40 °C. The mass spectrometer was operated in data-independent acquisition (DIA) mode with MS2 spectra acquired at 59 distinct 8 m/z shifted mass windows stepping from 436 to 900 m/z with a scan range of 140 to 1500 m/z, and an MS survey scan was obtained once every duty cycle from 430 to 915 m/z. MS, and MS2 spectra were acquired with an Orbitrap resolution of 70,000 and 30,000, respectively. The accumulation gain control (AGC) was set to 4 × 10^5^ and 5 × 10^5^ ions with a maximum injection time of 50 and 54 ms for MS and MS2 scans, respectively. The precursor ions in MS2 scans were selected across a mass isolation window of 12 m/z and fragmented by HCD (High Energy Collisional Dissociation) with a normalized collision energy of 30%.

### Untargeted Proteomics Data Analysis

Raw DIA mass spectra were analyzed with Protalizer micro-data independent acquisition (μDIA) software (v1.1.3154) from Vulcan Biosciences (24) after generating peak list data in mzML format with ProteoWizard MS convert (v3.0.11567). Overlapping MS2 spectra were deconvoluted and the mass-isolation windows reduced from 12 m/z to 4 m/z using a previously described approach with a 15 ppm fragment ion matching tolerance across MS2 scans (24). Peptide and protein identifications were made using the Protein Farmer peptide-centric search engine against the forward and reversed *mus musculus* Swiss-Prot database (2017-10 release) containing 16,704 sequences (not including decoys) with a 10 ppm precursor and fragment ion mass tolerance and a maximum expectation score of 0.05. All peptides were searched in the +2 and +3 precursor charge states, with corresponding y2–y15 and b2–b15 fragment ions in the +1 and +2 charge states. A minimum of seven fragment ions were required for all peptide-spectrum matches. Potential modifications searched for included oxidation of methionine residues, N-terminal protein acetylation, N-terminal protein methionine cleavage and acetylation, and pyro-glu of N-terminal glutamine and glutamic acid residues. Carbamidomethylation of cysteine residues was searched as a fixed modification. Only modifications unambiguously assigned to a specific amino acid site in a peptide were considered valid identifications. Peptides with ≤1 trypsin missed-cleavage were included in the analysis. In instances where different peptide ions (defined as having differences in either peptide sequence, precursor charge, or modification status) were matched to ≥1 fragment ions with m/z values ≥300 m/z in a particular MS2 scan, only a single peptide ion with the most significant expectation score was retained. Razor peptides corresponding to multiple proteins (including isoform versions of a protein) were assigned to a single protein entry in the *mus musculus* Swiss-Prot database with the most comprehensive sequence coverage. Proteins identified by a single razor peptide belonging to a group of proteins can be located in the identification files provided in the ProteomeXchange with the accession number PXD021884. A strict false discovery rate (FDR) of <1% for peptides and ≤5% for proteins was applied to each sample based on a reversed database search.

Lists of peptides identified by the search engine after FDR filtering were considered for matching to a corresponding MS2 chromatogram in every sample. Both y/b fragment ions with m/z values ≥300 assigned were used in MS2 chromatograms with a 15 ppm mass tolerance. The fragment ions belonging to a peptide MS chromatogram were required to be within >70% of their maximum intensity in any MS2 scan with the same precursor isolation window within ±20 s as described previously (24). The MS2 chromatogram intensities were calculated by determining the MS2 spectrum containing the largest sum intensity of all fragment ions assigned to a peptide ion and defining this as the peak apex, followed by summing the intensity of each fragment ion in this peak apex intensity scan as well as the two consecutive MS2 spectra preceding and following the apex intensity scan. To extract MS2 chromatograms corresponding to peptide ions that were not identified by the search engine in each sample, normalized retention times were calculated by a previously described approach using endogenous peptides without spiking-in retention time standards (25). With these estimated retention time coordinates, the MS2 chromatograms were extracted across all samples within ±20 s of the predicted elution time. Peptides assigned to MS2 chromatograms with ≥4 fragment ions were quantified. A local retention time intensity normalization approach was applied using endogenous peptides as described before (24).

Proteins were quantified using peptides universally matched to an MS2 chromatogram in all the samples compared with whenever >1 peptide existed for quantifying a protein as described previously (26). Missing values for peptide ions not quantified in a specific sample were assigned a zero value during statistical analysis. The normalized peptide amounts in each sample were placed in a relative fold-change scale with the smallest quantifiable amount in the samples compared assigned as “1.” From all the peptides assigned to the same protein entry, the average fold-change value was used as the protein fold-change per sample and Log_2_ transformed prior to statistical analysis by an unpaired *t* test.

Differentially abundant proteins meeting the following criteria (fold-change ≥ 1.3 and *p*-value ≤ 0.05) were uploaded to DAVID version 6.8 (27, 28) and subjected to pathway analysis for terms in the Kyoto Encyclopedia of Genes and Genomes (KEGG) pathway database. The reference background used for pathway analysis was chosen to be all the quantified proteins in the untargeted proteomics dataset (27). A modified Fisher’s exact test (*i.e.*, EASE score) was used to determine the significance of enriched pathways (28), without applying a multiple testing correction technique (29, 30), and only pathways with *p*-value <0.01 were considered valid.

### Development and Analytical Validation of Targeted MS Measurements

A tier 3 targeted proteomics assay was used to verify the quantification of a select panel of proteins by a parallel reacting monitoring mass spectrometry (PRM-MS) approach adapted from Peterson *et al.* (31). From each sample, 50 μg of protein in 8 M urea/100 mM Tris-HCl pH 8.3 was proteolytically digested in-solution with sequencing grade Lys-C/trypsin (Promega) and (enzyme:protein) ratio (w/w) of 1:25. Digestions were carried out for 4 h at 37 °C, then the samples were diluted tenfold to 0.8 M urea with 50 mM Tris-HCl pH 7.5 and digested another 16 h at 37 °C. The digested peptides were acidified with trifluoroacetic acid (TFA) to a final concentration of 0.5% (v/v), desalted with C18 resin Zip Tips (EMD Millipore), eluted with 60% acetonitrile/0.1% TFA, lyophilized in a vacuum centrifuge, and reconstituted in 2% acetonitrile/0.1% TFA for LC-MS/MS analysis. The final peptide concentrations were measured using a fluorometric peptide assay (Pierce) prior to PRM-MS analysis. From each sample 1.2 μg was separated on an EASY-nLC 1200 UHPLC with a custom Proxeon nanospray source and the column oven heated to 35 °C. Peptides were loaded onto a 100 μm × 25 mm Magic C18 100 Å 5 μm reverse-phase trap before being separated with a 75 μm × 150 mm Magic C18 200 Å 3 μm reverse-phase column packed in house. Peptides were eluted by an increasing percentage of acetonitrile over a 90 min analytical gradient with a flow rate of 300 nl/min. Mass spectra were acquired on a Q-Exactive Plus instrument (Thermo Scientific) in positive ion mode, with the PRM method set to trigger an inclusion list of 30 target peptide masses without retention time scheduling. MS2 spectra for each target mass were obtained with a resolution of 17,500, an AGC target of 5 × 10^5^ ions, and maximum injection time of 80 ms. A mass isolation window of 1.6 *m/z* was used for precursor ion selection, with a fixed first mass of 140 *m/z*, and HCD normalized collision energy of 25%. For each biological replicate, two PRM LC-MS/MS runs were performed with a separate list of 30 target masses (for a total of 60 target masses/sample). The average duty cycle was 3.2 s with both acquisition lists providing sufficient peak shape determination with approximately 5 to 10 MS2 scans during the elution width of each peptide.

For each targeted protein, three proteotypic peptides were included in the inclusion list from the untargeted proteomic dataset with the most intense average MS2 chromatograms and that were unique to the targeted protein in the *mus musculus* Swiss-Prot reference proteome (2017-10 release). An exception to the latter requirement was Acot1 where the peptide SEFYADEISK is also present in the Acot2 isoform. In cases where a specific protein lacked three unique and detectable peptides in the untargeted proteomics dataset, additional peptides unique to the targeted protein were selected from the SRM atlas (32) (supplemental Table S1 has all the peptides targeted by PRM). Protalizer PRM software (v1.1.3966) was used to analyze all PRM-MS mass spectra after generating peak lists in mzML format with ProteoWizard MS convert (v3.0.11567). MS2 chromatograms were extracted from the targeted peptides with ≥6 y2–y15 and b2–b15 ions in the +1 or +2 charge state with a mass tolerance of 20 ppm. All the transitions were required to have a similar apex intensity retention time by eliminating fragment ions not within >70% of their maximum intensity in any MS2 scan with the same precursor isolation window within ±20 s as described previously (24). Peptide quantification across samples was performed by MS2 chromatogram intensities and required ≥four transitions with m/z values ≥300 (low m/z values were removed to eliminate interferences in the measurements). Proteins were quantified by peptides universally matched to an MS2 chromatogram in all the samples compared with whenever >1 peptide was available for quantifying a protein as described earlier (26). Missing values for peptide ions not quantified in a specific sample were assigned a zero value during statistical analysis. The MS2 chromatogram intensity was summed with all the other peptides belonging to the same target protein, then normalized to the total ion current (TIC) from each sample, Log_2_ transformed, and subjected to an unpaired *t* test for statistical analysis.

### Experimental Design and Statistical Rationale

In the untargeted proteomics and PRM-MS experiments, four biological replicates were analyzed in randomized order for both the *GFAP*^*Tg*^;*Gfap*^+/*R236H*^ and wild-type mice. The control samples used in all experiments were age-matched 25-day-old wild-type mice. Data analysis was performed as described above and excel was used for statistical analyses. No outlier removal steps were applied to any peptides or proteins in the untargeted proteomics and PRM datasets. The significance criteria used for pathway analysis of differentially abundant proteins (see above) were chosen based on a prior study of quantification performance with spiked-in proteins in complex mixtures (24).

## Results

### Overall Proteomic Experimental Findings

**Fig. 1:**
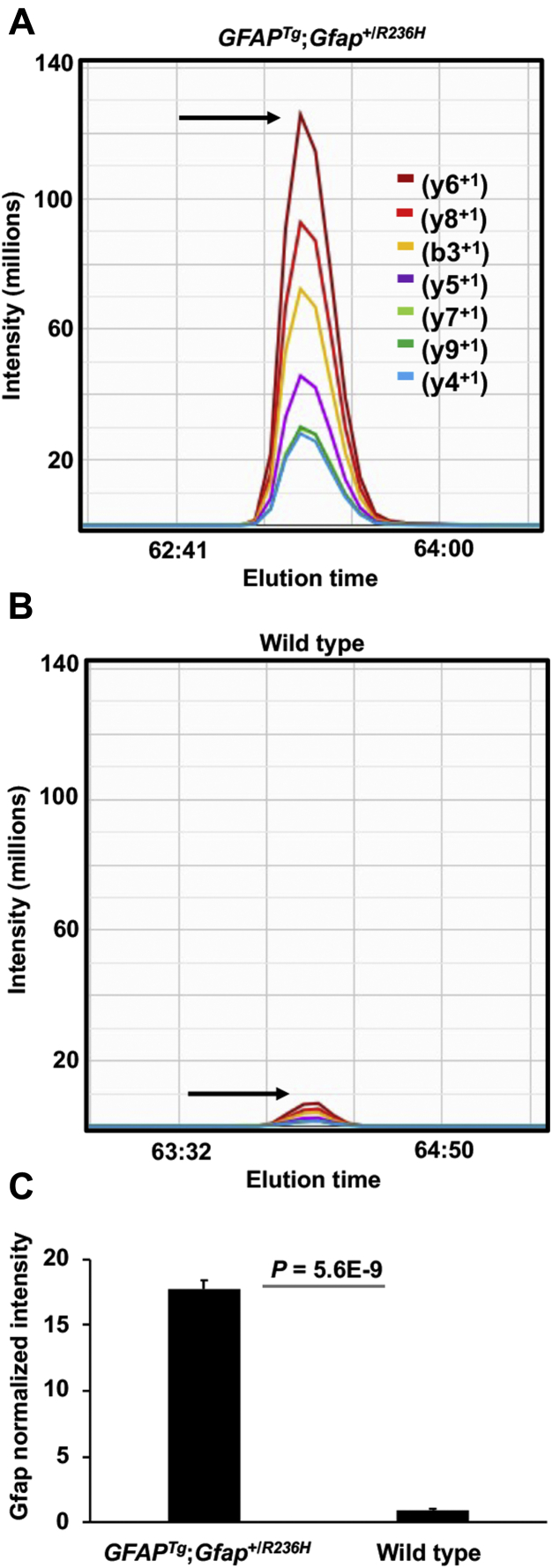
**Elevation of GFAP in the *GFAP***^***Tg***^**;*Gfap***^**+/*R236H***^**mice quantified by μDIA mass spectrometry.***A* and *B*, representative Gfap MS2 chromatograms are shown for the peptide sequence LADVYQAELR, which is present in both mouse Gfap (residues 109–118) and human GFAP (residues 112–121). The *arrow* points to the peptide apex intensity in each chromatogram. *A*, shows the peptide is more abundant in *GFAP*^*Tg*^;*Gfap*^+/*R236H*^ mice compared with (*B*), which shows a wild-type animal. *C*, average normalized intensity of GFAP across 37 peptides in the *GFAP*^*Tg*^;*Gfap*^+/*R236H*^ and wild-type mice. Error bars are standard deviation (stdev).

The clinical symptoms of AxD patients are heterogeneous with anatomical abnormalities occurring in multiple brain regions (13). Therefore, whole brains were analyzed in this study from *GFAP*^*Tg*^;*Gfap*^+/*R236H*^ mice at 25 days of age when these animals have severe gliosis and RF formation (23). Consistent with the induction of gliosis in the brain tissues analyzed from *GFAP*^*Tg*^;*Gfap*^+/*R236H*^ mice, the levels of GFAP were significantly upregulated at this timepoint (Fig. 1).

In total, the untargeted proteomics experiment quantified the relative abundance of 5005 proteins through the identification of 52,378 unique peptide ions. The average coefficient of variation (CV) for all the peptides quantified in the *GFAP*^*Tg*^;*Gfap*^+/*R236H*^ and wild-type mice was 15.2% and 15.7%, respectively, indicating the measurements were highly reproducible across biological replicates (n = 4 per genotype). There were 635 statistically significant protein changes found between the *GFAP*^*Tg*^;*Gfap*^+/*R236H*^ and wild-type mice (*p*-value ≤ 0.05 and a fold-change ≥ 1.3 or fold-change ≤ −1.3 in *GFAP*^*Tg*^;*Gfap*^+/*R236H*^ *versus* wild-type mice, supplemental Table S2).

### Analysis of Prior Findings Related to AxD

**Fig. 2:**
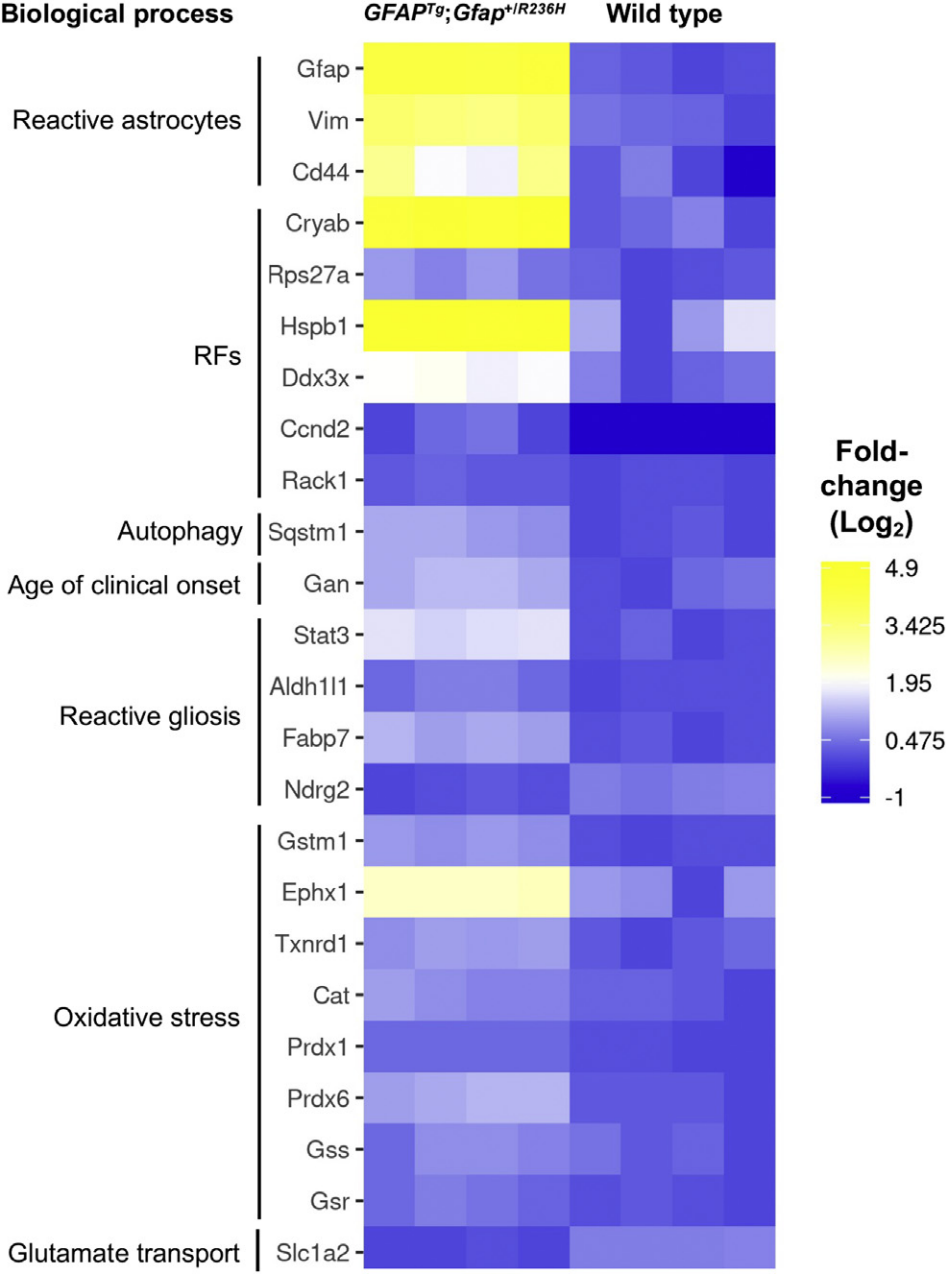
**RF and reactive gliosis proteins are among the most changed in the quantified proteome of *GFAP***^***Tg***^**;*Gfap***^**+/*R236H***^**mice *versus* wild type.** Heat map of proteins previously documented in AxD to have altered abundance, as well as three reactive gliosis proteins (Fabp7, Aldh1l1, Ndrg2) that have not previously been linked to AxD phenotype. Data shown are the individual mouse Log2 normalized protein fold-change values in every sample. Proteins without detectable amounts in a particular sample were assigned a Log_2_ normalized fold-change value of −1 (*dark blue* in the heat map).

*GFAP*^*Tg*^;*Gfap*^+/*R236H*^ mice recapitulate neuropathological features observed in human patients with AxD, including reactive gliosis, the presence of ubiquitinated RFs, activation of stress-response pathways, attenuated levels of the glutamate transporter in astrocytes, and induction of autophagy (Fig. 2). Consistent with these previous reports, increases were detected in proteins associated with reactive astrocytes (Gfap, Vim, Cd44) (23), RFs (Cryab, Rps27a, Hsp27 (also known as Hspb1), Ddx3x, Ccnd2, Rack1) (22, 33, 34), and autophagy (Sqstm1) (35). Gan, which targets Gfap for proteasomal degradation (36), was also elevated and has gene variants correlated with the age of clinical onset in AxD patients (37). Heightened levels of Stat3 were also detected in *GFAP*^*Tg*^;*Gfap*^+/*R236H*^ mice, a phosphorylated transcription factor activated in reactive gliosis (38). Several protein differences previously associated with reactive astrocytes, but not yet specifically reported in AxD, were also observed. These include increased abundance of Aldh1l1 (39) and Fabp7 (40) and a decrease in Ndrg2 (41). Reflective of the increased oxidative stress in *GFAP*^*Tg*^;*Gfap*^+/*R236H*^ mice, there was pronounced upregulation of the Nfe2l2-mediated stress response (Gstm1, Ephx1, Txnrd1, Cat, Prdx1, Prdx6, Gss, and Gsr) (19, 20) and diminished levels of the astrocyte glutamate transporter (Slc1a2, also known as Glt-1) (15, 16, 18, 23). Collectively, these findings validate the untargeted proteomics dataset and expected molecular changes in the brains of *GFAP*^*Tg*^;*Gfap*^+/*R236H*^ mice.

### Altered Biological Pathways

**Fig. 3:**
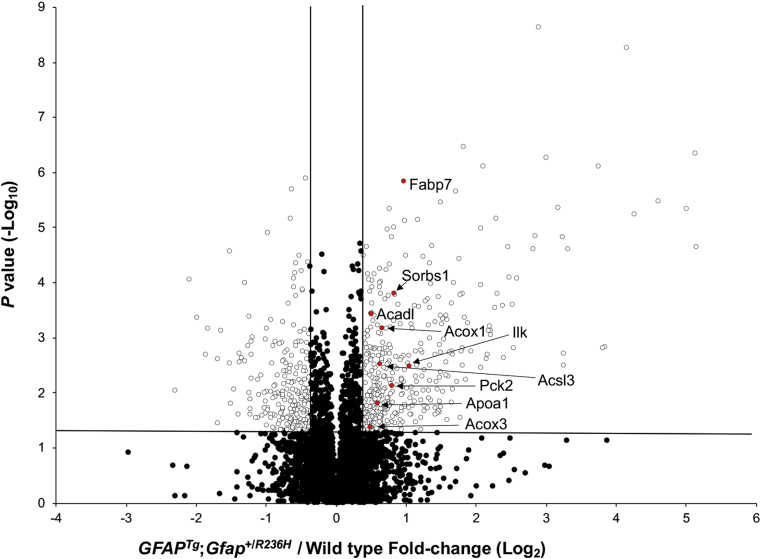
**PPAR pathway proteins are elevated in *GFAP***^***Tg***^**;*Gfap***^**+/*R236H***^**mice.** Data shown are untargeted μDIA proteomic quantification results of 4938 proteins (67 proteins that are listed in supplemental Table S2 but detected only in the *GFAP*^*Tg*^;*Gfap*^+/*R236H*^ or wild-type mice are not included in this plot). PPAR pathway proteins are highlighted in red with Swiss-Prot gene abbreviations. The vertical lines denote fold-change values of −1.3 and +1.3 (without Log transformation), and the horizontal line represents a *p*-value = 0.05 (without Log transformation).

To detect signaling cascades perturbed in *GFAP*^*Tg*^;*Gfap*^+/*R236H*^ mice, the list of proteins having significant quantitative differences between the AxD model mice and controls was analyzed with the online DAVID tool against the KEGG database. Two pathways were identified as upregulated, glutathione metabolism, which has been previously implicated in AxD (19, 20), and the PPAR signaling pathway, which represents a novel finding (Table 1; Fig. 3). PPARs (peroxisome proliferator-activated receptors) are nuclear hormone receptors that regulate the metabolism of lipids and are activated by prostaglandins as well as fatty acids and related molecules such as lysophosphatidic acid (LPA) (42). To investigate which of the three PPAR isotypes (PPARα, PPARβ/δ, and PPARγ) might be involved, we next searched our dataset for proteins reported to be regulated by particular PPAR isotypes. The results of this search suggested involvement of both PPARα signaling due to increased levels of (Pnpla2, Acadvl, Acot1, Acsl3, Hmgcs2, Acox1) (43, 44, 45) and PPARγ signaling (Sorbs1) (46). Additionally, the protein levels of Apoa1 were also elevated, which has been reported to be increased by PPARα (47) and PPARγ (48). No significantly altered canonical pathways were found using the list of proteins decreased in the *GFAP*^*Tg*^;*Gfap*^+/*R236H*^ mice (supplemental Table S2).

**Table 1:** Differentially regulated protein signaling pathways in *GFAP*^*Tg*^;*Gfap*^+/*R236H*^ mice

Upregulated pathways	Increased proteins in the pathway	*p*-value
Glutathione metabolism	Gsta3, Gstm1, Gclc, Gpx4, Pgd, Gclm, Gss, Gsto1, Gpx3, Gpx1, Idh2	1.47E-04
PPAR signaling	Ilk, Fabp7, Sorbs1, Apoa1, Acadl, Acsl3, Acox3, Pck2, Acox1	2.75E-03

Pathways assigned a *p*-value ≤ 0.01 by the DAVID analysis tool are displayed. Protein abbreviations shown correspond to Swiss-Prot genes. No significant pathways using the list of downregulated proteins were observed.

### Resolution of Proteins Associated with Brain Cell Types

To determine CNS cell types contributing to pathology in *GFAP*^*Tg*^;*Gfap*^+/*R236H*^ mice, a database of acutely isolated cell-type enriched proteins in mouse brain (49) was cross-referenced with the differentially regulated proteins from the whole brain tissue analysis in this study. The results of this analysis are described below.

### Astrocyte-Related Proteins Altered in *GFAP*^*Tg*^;*Gfap*^+/*R236H*^ Mice

AxD is nearly unique among neurological disorders because the primary genetic defect of the disorder originates in astrocytes, with the only known exception to this being vanishing white matter disease (50). Astrocytes have a central role in brain metabolism and shuttle lactate from glycolysis to neurons (3) in response to physiological processes such as learning and memory (51) or injury (52). With respect to enzymes involved in glycolysis, the *GFAP*^*Tg*^;*Gfap*^+/*R236H*^ mice display possible signs of increased accumulation of glycogen due to exhibiting decreases in glycogen phosphorylase (Pygb) and increased levels of the glucosyl donor (Ugp2).

Fatty acid metabolism is enriched in astrocytes relative to other brain cells (53), and multiple proteins associated with this process were found altered in the untargeted proteomics analysis. For instance, *GFAP*^*Tg*^;*Gfap*^+/*R236H*^ mice have increased levels of the long-chain fatty acid transporter Slc27a1 and the brain isoform of fatty acid-binding protein, Fabp7. *GFAP*^*Tg*^;*Gfap*^+/*R236H*^ mice also have increased amounts of proteins consistent with elevated concentrations of fatty acids; *e.g.*, increased acyl-coenzyme A thioesterase 1 (Acot1), in conjunction with decreases in long-chain-fatty-acid--CoA ligase 6 (Acsl6). However, there were decreased amounts of the enzyme responsible for *de novo* synthesis of fatty acids, Fasn, suggesting that astrocytes in *GFAP*^*Tg*^;*Gfap*^+/*R236H*^ mice have increased utilization of exogenous fatty acids.

Astrocytes are also key regulators of glutamate metabolism in the brain (54). As already mentioned, we observed decreased amounts of the glutamate uptake transporter Slc1a2, as previously reported in the *GFAP*^*Tg*^;*Gfap*^+/*R236H*^ mice (15, 16, 18, 23). Additionally, the untargeted proteomics analysis identified decreases in the transporter responsible for the efflux of glutamine from astrocytes, Slc38a3 (55), and in the levels of the mitochondrial glutamate carrier Slc25a18.

Differences in mitochondrial metabolism is suggested in the *GFAP*^*Tg*^;*Gfap*^+/*R236H*^ mice by a decrease in the mitochondrial citrate transporter Sfxn5. Additionally, increased levels were found for several mitochondrial proteins involved in the generation of NADPH, including Idh2 and Nadk2, as well as Pgd which participates in NADPH generation *via* the pentose phosphate pathway (PPP). Due to the extensive degree of oxidative stress in the *GFAP*^*Tg*^;*Gfap*^+/*R236H*^ mice (18), any increases in NADPH may be more relevant for powering redox defense than providing reducing equivalents for macromolecular biosynthesis (56).

Differentially abundant astrocyte-related proteins were also annotated due to their function as ion channels or cell membrane proteins. Most notably, the *GFAP*^*Tg*^;*Gfap*^+/*R236H*^ mice had lower amounts of the electrogenic sodium bicarbonate cotransporter Slc4a4, a high-affinity bicarbonate carrier expressed in cortical astrocytes (57). This Slc4a4 reduction, coupled with deficits in the carbonic anhydrase, Ca2, suggests that astrocytes in *GFAP*^*Tg*^;*Gfap*^+/*R236H*^ mice may undergo acidification. This could stimulate toxic release of glutamate, a process that occurs when astrocyte acidosis is caused by ischemic injury (58). There were also decreases in the sodium- and chloride-dependent GABA transporter Slc6a11, which may alter the epileptic activity observed in AxD (59, 60). Decreases in several additional ion channels were also found whose function is less well characterized in astrocytes, such as the chloride channel protein tweety Ttyh1 and the beta subunit of the sodium/potassium-transporting ATPase Atp1b2. Last, there were decreased amounts of the Gpr37l1 receptor that binds prosaposin and reduces oxidative stress in astrocytes when stimulated (61, 62). The deficit in Gpr37l1 may cause detrimental effects in *GFAP*^*Tg*^;*Gfap*^+/*R236H*^ mice, given the pronounced level of oxidative stress in the brain white matter of these mice (18).

Various astrocyte functions are attributable to tightly regulated proteins that increase or decrease during the maturation of these cells. By cross-referencing an RNA-seq database (63) containing markers of nascent *versus* mature astrocytes (mouse P7 *versus* P32 astrocytes and fetal *versus* mature human astrocytes), numerous proteins, which increase during healthy maturation, were found decreased in P25 *GFAP*^*Tg*^;*Gfap*^+/*R236H*^ mice compared with their wild-type controls (Slc1a2, Gja1, Mlc1, Kcnj10, Slc38a3, S1pr1, Sparcl1). Notably, these developmentally deficient proteins are capable of altering other cell types due to being membrane-associated proteins or a secreted factor in the case of Sparcl1.

### Findings Associated With Other Brain Cell Types

**Fig. 4:**
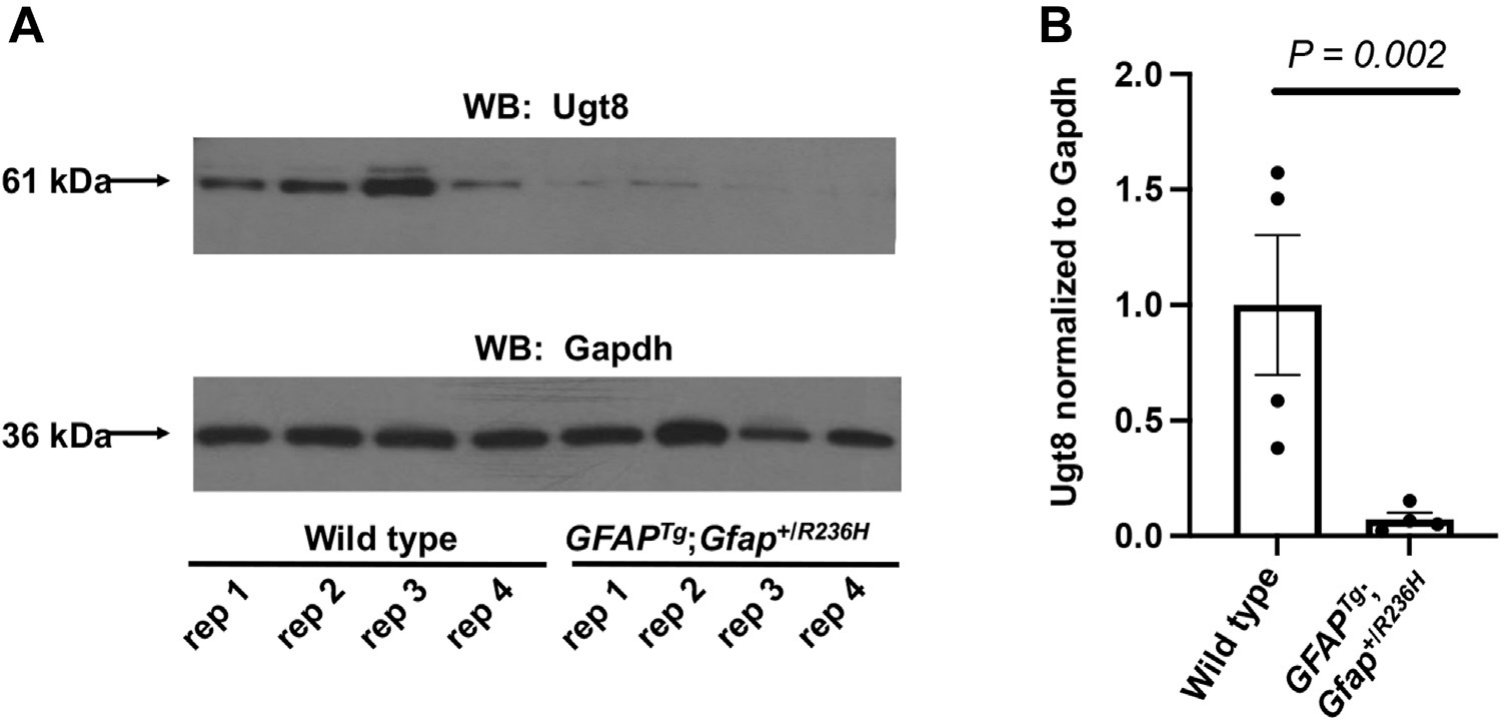
**Ugt8 is decreased in *GFAP***^***Tg***^**;*Gfap***^**+/*R236H***^**mice.***A*, Western blot of Ugt8 and Gapdh (loading control). Data from the molecular weight ladder is not shown. Each (rep) is a biological replicate from each genotype. *B*, average Western blot fold-change of Ugt8 normalized to Gapdh in the *GFAP*^*Tg*^;*Gfap*^+/*R236H*^ mice relative to wild type was −13.7 fold. Error bars are standard error of the mean (SEM).

Reactive astrocytes secrete factors that block oligodendrocyte maturation (64), thereby inhibiting myelination in the context of various white matter injuries (65). Accordingly, it was next assessed whether *GFAP*^*Tg*^;*Gfap*^+/*R236H*^ mice exert effects on proteins preferentially expressed in oligodendrocytes. Consistent with a previous report that the transcripts of myelin-related genes are downregulated in the hippocampus of *GFAP*^*Tg*^;*Gfap*^+/*R236H*^ mice (19), decreases were found in the classical protein constituents of myelin (Cnp, Mbp, Mog, Mobp, Mag, and Plp1) (Table 2). Of particular interest, the level of UDP-galactose-ceramide galactosyltransferase (Ugt8), a regulator of myelin membrane synthesis, was profoundly decreased. Similar to *GFAP*^*Tg*^;*Gfap*^+/*R236H*^ mice, *Ugt8*-deficient mice form myelin that also appears ultrastructurally normal and exhibit similar phenotypes as the *GFAP*^*Tg*^;*Gfap*^+/*R236H*^ mice, including a generalized tremor, mild ataxia, and age-related hindlimb paralysis (66). Because the protein levels of Ugt8 in the whole brain specimens of *GFAP*^*Tg*^;*Gfap*^+/*R236H*^ mice suggested a far more substantial decrease than reported in hippocampus transcripts (*i.e.*, hippocampus transcripts were decreased −1.7-fold in ref. (19), *versus* −4.3-fold in the whole brain proteomic analysis), the deficit in Ugt8 was further examined by Western blotting (Fig. 4). These results confirmed that Ugt8 is markedly deficient in the *GFAP*^*Tg*^;*Gfap*^+/*R236H*^ mice, yielding an average decrease of −13.7-fold. Additionally, the observed deficits in the classical constituents of myelin in *GFAP*^*Tg*^;*Gfap*^+/*R236H*^ mice may be related to another astrocyte-secreted factor, chitinase-like protein 3 (Chil3), that was found upregulated in *GFAP*^*Tg*^;*Gfap*^+/*R236H*^ mice. For example, another chitinase-related protein with 39.6% sequence identity to Chil3, Ch3l1 also known as Ykl-40, inhibits myelination by effecting oligodendrocyte precursors in AxD models created from human patient induced pluripotent stem cells (67).

**Table 2:** LC-MS/MS results showing downregulation of oligodendrocyte-lineage proteins are generally consistent with previously reported RNA profiles

Accession	Gene	Protein	μDIA fold-change (*GFAP^Tg^;Gfap^+/R236H^*/wild type)	RNA-seq (*GFAP^Tg^;Gfap^+/R236H^*/wild type)
Q64676	Ugt8	2-hydroxyacylsphingosine 1-beta-galactosyltransferase	−4.28∗∗	−1.72∗
P16330	Cnp	2′,3′-cyclic-nucleotide 3′-phosphodiesterase	−1.31∗∗	−1.55∗∗
Q61885	Mog	Myelin-oligodendrocyte glycoprotein	−1.21∗∗	−1.45
P04370	Mbp	Myelin basic protein	−1.46∗	−1.40∗
Q9D2P8	Mobp	Myelin-associated oligodendrocyte basic protein	−1.28	−1.49∗∗
P60202	Plp1	Myelin proteolipid protein	−1.33∗	−1.52
P20917	Mag	Myelin-associated glycoprotein	−1.35∗∗	−1.40∗

The average fold-change protein ratio measurements for *GFAP*^*Tg*^;*Gfap*^+/*R236H*^/wild-type mice shown were collected by untargeted proteomics (*i.e.*, μDIA LC-MS/MS). Transcript measurements were taken from previously published RNA-seq data (19). Statistical analysis in both datasets is from a two-tailed *t* test (∗∗*p* < 0.01, ∗*p* < 0.05).

Prior reports from AxD patient brain documented neuronal loss in the hippocampus and striatum (15). Additionally, the earlier hippocampus transcriptome analysis of *GFAP*^*Tg*^;*Gfap*^+/*R236H*^ mice reported relatively small decreases of ∼20% in several synaptic proteins (Snap25, Dlg4, Bsn) with highly specific neuronal expression (19). However, in the analysis of whole brain protein lysates from *GFAP*^*Tg*^;*Gfap*^+/*R236H*^ mice, no decreases were found in these proteins. This discrepancy maybe due to surveying the entire brain rather than a discrete region of the CNS or because RNA levels are not always directly correlated to protein concentration (21).

*GFAP*^*Tg*^;*Gfap*^+/*R236H*^ mice exhibit a marked inflammatory response with microglial activation (19). In agreement with this, cathepsin D (Ctsd), a microglia-enriched protein, was increased in the *GFAP*^*Tg*^;*Gfap*^+/*R236H*^ mice, as well as complement proteins secreted by microglia (C1qa, C1qb, C1qc), which are capable of inducing reactive astrogliosis (68, 69).

### PRM-MS Confirmation of Protein Differences

PRM-MS was employed to validate a targeted list of proteins lacking a prior association to AxD and with altered amounts in the *GFAP*^*Tg*^;*Gfap*^+/*R236H*^ *versus* wild-type mice. Specifically, proteins were validated due to having a close association to astrocytes (Fabp7, Slc6a11, Slc4a4, Slc38a3, Ca2, Slc25a18, Gpr37l1, Nadk2, Sfxn5), fatty acid metabolism in astrocytes (Fasn, Slc27a1, Acot1, Acsl6), oligodendrocytes (Ugt8, Mbp), and the BDNF/NT-3 growth factors receptor Ntrk2 expressed in neurons and astrocytes (49) (Table 3). Only one of these, Ntrk2 was not detected in the PRM assay; the other 15 proteins had relative changes in the same direction as found in the untargeted μDIA proteomic analysis, with 13 of these changes having statistical significance (*p*-value ≤ 0.05) (supplemental Table S3 contains all the targeted peptide quantification results from the PRM experiment with CV values). Lastly, four proteins found to be unchanged in the untargeted μDIA proteomics dataset served as negative controls and were also found to be unchanged by PRM-MS (Table 3). All of the MS2 chromatograms for the PRM experimental results in this study are available in HTML format for visualization (supplemental Figs. S1 and S2).

**Table 3:** Verification of μDIA untargeted proteomics quantification by targeted PRM-MS

Gene	μDIA fold-change	PRM fold-change	*p*-value μDIA	*p*-value PRM
Fabp7	1.95	1.88	1.47E-06	3.13E-03
Slc6a11	−1.57	−2.44	6.96E-06	2.64E-04
Slc4a4	−1.43	−2.16	3.36E-05	1.23E-02
Slc38a3	−1.52	−1.29	1.27E-04	1.37E-01
Gpr37l1	−1.67	downregulated	2.60E-03	2.29E-06
Nadk2	2.58	2.57	2.24E-05	6.07E-04
Sfxn5	−1.38	−1.38	8.63E-03	1.40E-03
Fasn	−1.49	−1.68	4.68E-05	6.67E-03
Ugt8	−4.28	−3.71	9.16E-05	2.74E-03
Mbp	−1.46	−2.16	1.67E-02	7.17E-03
Ntrk2	−1.74	not detected	2.48E-02	not detected
Slc27a1	1.41	1.94	8.11E-02	1.67E-02
Acot1	1.53	1.43	8.26E-04	1.49E-02
Acsl6	−1.49	−1.88	1.46E-03	4.91E-02
Slc25a18	−1.55	−1.20	2.24E-03	4.16E-01
Ca2	−1.31	−1.41	5.37E-03	3.45E-02
∗Slc6a17	−1.07	−1.45	2.09E-01	2.44E-01
∗Stxbp5l	−1.16	−1.17	1.17E-01	4.59E-01
∗Cryl1	1.18	−1.14	2.56E-01	4.88E-01
∗Slc16a1	−1.15	−1.15	1.44E-01	1.49E-01

Comparison of the average fold-change ratio of proteins in (*GFAP*^*Tg*^;*Gfap*^+/*R236H*^/wild-type mice) and associated *p*-values in the μDIA *versus* PRM-MS experiments for 20 proteins selected for confirmation (n = 4 per genotype in both experiments). (∗) indicates proteins designated as negative controls with insignificant *p*-values from μDIA untargeted proteomics and PRM-MS. The “downregulated” result indicates the protein was only detectable in wild-type mice.

### Neuroanatomical Distribution of Fabp7 and Ugt8 in *GFAP*^*Tg*^;*Gfap*^+/*R236H*^ Mice

**Fig. 5:**
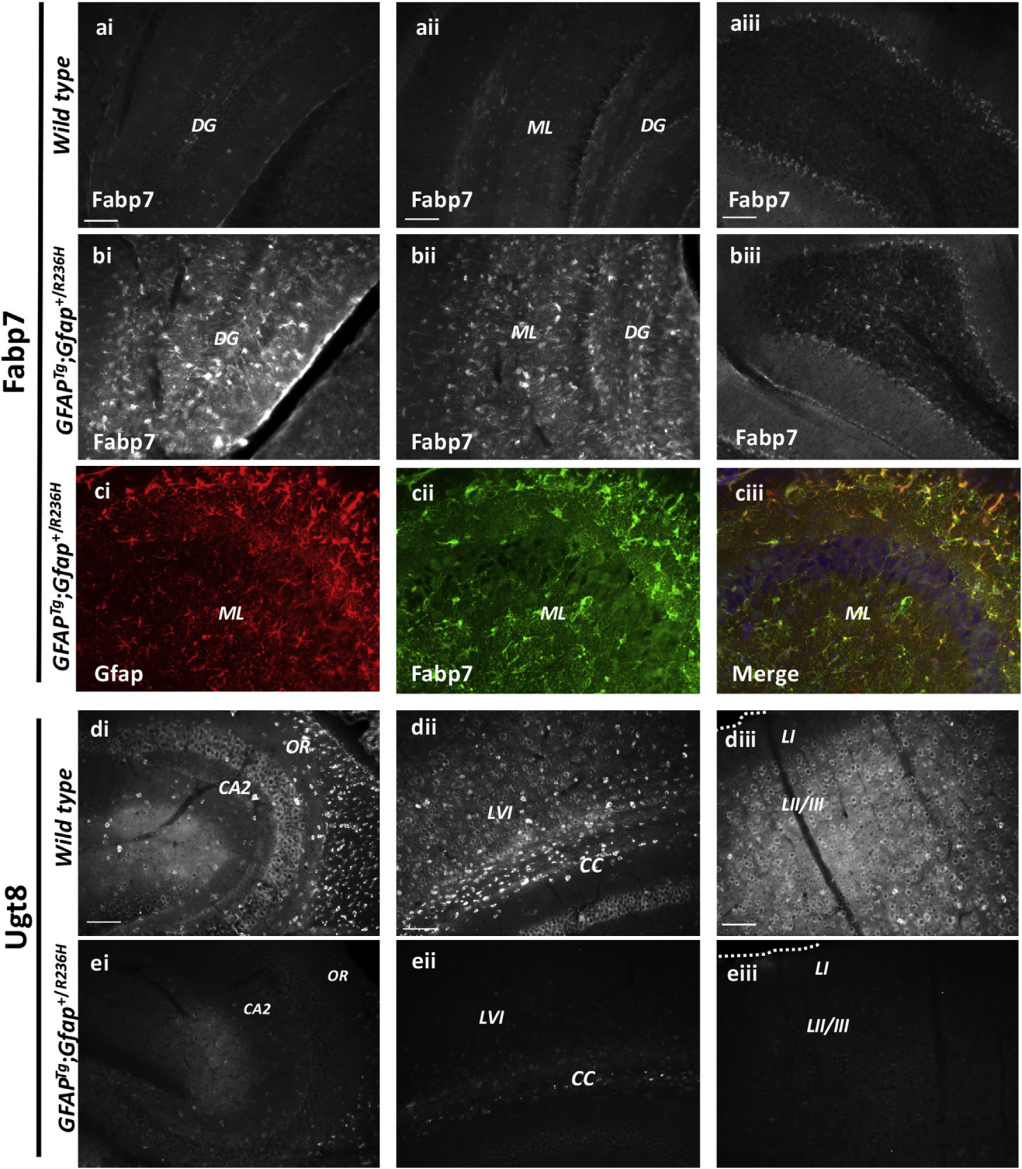
**Brain region alterations in Fabp7 and Ugt8 in wild type compared with *GFAP***^***Tg***^**;*Gfap***^**+/*R236H***^**mice.***A* and *B*, Fabp7 immunoreactivity is increased in *GFAP*^*Tg*^;*Gfap*^+/*R236H*^ hippocampal regions relative to the same regions in wild-type littermates. The fifth cerebellar lobe (5 CB) is shown for reference. *C*, double immunolabeling with Gfap (*C*i, *red*) and Fabp7 (*C*ii, *green*) indicates significant colocalization in the merged image (*C*iii, merge, DAPI- a nuclear stain is shown in *blue*). Wild-type mice (*A*i–*A*iii) and *GFAP*^*Tg*^;*Gfap*^+/*R236H*^ mice (*B*i–*C*iii). *D* and *E*, Ugt8 immunoreactivity is decreased across the hippocampus (*E*i), corpus callosum (*E*ii), and cortex (*E*iii) in *GFAP*^*Tg*^;*Gfap*^+/*R236H*^ mice relative to wild-type littermates (*D*i–*D*iii) (Scale bar = 100 μm). CA2, CA2 hippocampal region; CC, corpus callosum; DG, dentate gyrus; LI, LII/III and LVI, cortical layers 1, 2/3, and 6; ML, molecular layer; OR, oriens layers.

Fabp7, a protein that plays a central role in reactive gliosis (40), and was found elevated in *GFAP*^*Tg*^;*Gfap*^+/*R236H*^ mice, was next analyzed for brain region differences (Fig. 5, *A*i–*C*iii) since the initial μDIA untargeted proteomics analysis was performed on whole brain protein homogenates. Additionally, Ugt8 was analyzed for brain region differences in protein expression (Fig. 5, *D*i–*E*iii), due to being decreased in *GFAP*^*Tg*^;*Gfap*^+/*R236H*^ mice and a critical enzyme in the synthesis of the myelin lipids galactocerebroside and sulfatide (66). Fabp7 immunoreactivity was markedly upregulated in all brain regions, but the most robust increases were observed in the hippocampal regions of *GFAP*^*Tg*^;*Gfap*^+/*R236H*^ mice (Fig. 5, *B*i and *B*ii). The fifth cerebellar lobe (Fig. 5, *A*iii–*B*iii), which had a more modest increase in Fabp7, is also shown. Double immunofluorescent labeling for the intermediate filament Gfap (Fig. 5*C*i, red) indicates that Fabp7 (Fig. 5*C*ii, green) is highly expressed in most hippocampal Gfap^+^ cells (Fig. 5*C*iii, merged image), similar to results from cortical stab injury models where Fabp7 is also markedly upregulated in Gfap^+^ cells (40). Alternatively, Ugt8 exhibited marked reductions in immunoreactivity in the *GFAP*^*Tg*^;*Gfap*^+/*R236H*^ mice relative to wild-type animals in all brain regions examined, which included the hippocampus (Fig. 5, *D*i–*E*i), corpus callosum (Fig. 5, *D*ii–*E*ii), and cortical layers I-III (Fig. 5, *D*iii–*E*iii). In wild-type mice, nonastrocyte cells lacking Gfap colocalization (data not shown) had low levels of perinuclear labeling of Ugt8, but high levels of immunoreactivity in small cell bodies throughout the gray matter and in the corpus callosum (Fig. 5, *D*i–*D*iii).

### Comparison of FABP7 and UGT8 in AxD Patients and Human Controls

**Fig. 6:**
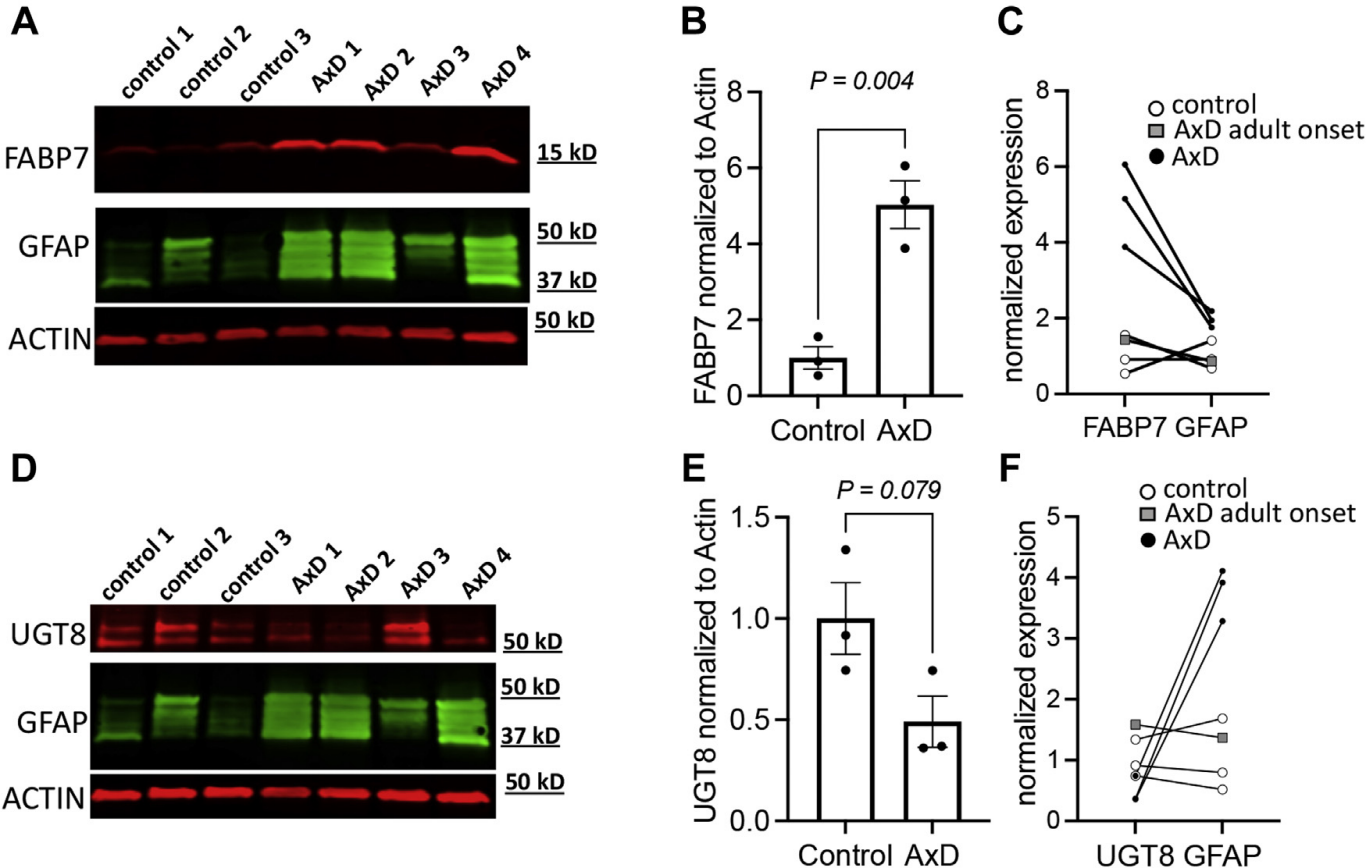
**UGT8, FABP7, and GFAP Western blotting in AxD patient and human controls.***A*–*F*, in all Western blots the samples loaded in each lane from *left*-to-*right* were: lane 1 is a 15-year-old male control, lane 2 is a 50-year-old female control, lane 3 is a 1-year-old male control, lane 4 is a 6-year-old male R239C AxD patient, lane 5 is a 14-year-old male R79C AxD patient, lane 6 is a 50-year-old female S247P AxD patient, and lane 7 is a 1-year-old male R239H AxD patient. Additional demographics for these samples are provided in supplemental Table S4. *A*, Western blot of FABP7, ACTIN (loading control), and GFAP. *B*, average Western blot fold-change for FABP7 normalized to actin with adult-onset patient sample removed (error bars SEM). *C*, relationship between FABP7 and GFAP for each control and AxD tissue sample. *D*, Western blot of UGT8, actin (loading control), and GFAP. *E*, average Western blot fold-change for UGT8 normalized to actin with adult-onset patient sample removed (error bars SEM). *F*, relationship between UGT8 and GFAP for each control and AxD tissue sample.

Due to the low frequency of AxD in human live births (14) and scarcity in AxD patient brain tissue, it is not possible to perform human brain tissue analyses alone that have adequate statistical power to obtain definitive findings. Nevertheless, prior studies have reported using a combination of AxD mouse models and a limited number of human AxD patient analyses can provide verification that the findings in *GFAP*^*Tg*^;*Gfap*^+/*R236H*^ mice are relevant to the human disease (22). Therefore, to determine whether the elevation of FABP7 and downregulation of UGT8 also occurs in the human disorder, Western blotting was performed in AxD patient (n = 4) relative to control (n = 3) brain tissue homogenates (supplemental Table S4 has sample demographic information and normalized protein values for FABP7 and UGT8). A GFAP Western blot was also performed to enable comparison to an established AxD biomarker. In accordance with the results from the *GFAP*^*Tg*^;*Gfap*^+/*R236H*^ mice, results indicated that for the samples obtained from individuals with Type I or early onset AxD *versus* control samples, an increase in FABP7 and a trend toward a decrease of UGT8 in cortical tissue (Fig. 6). As indicated above, AxD can be classified by two different forms, Type I and Type II. Type I is typically early onset (<4 years of age) with cortical involvement, developmental delay, spasticity, and seizures with a median survival of about 14 years (14). In our small human tissue cohort, lanes 4, 5, and 7 correspond to male individuals which died from AxD by 14 years of age. These cortical tissue samples demonstrate high GFAP levels. Type II onset can occur at any age and has a median survival of 25 years. Common symptoms of Type II differ from Type I, including autonomic dysfunction and bulbar-like symptoms (14). Individuals with Type II AxD are often negative for neurocognitive or developmental deficits. The value for the AxD patient that appears similar to control samples corresponded to the 50-year-old adult onset female S247P sample. Notably, this sample was similar to control samples for FABP7, UGT8, and GFAP (Fig. 6, *C* and *F*, gray boxes), consistent with the reduced amount of cortical involvement reported for Type II AxD.

**Fig. 7:**
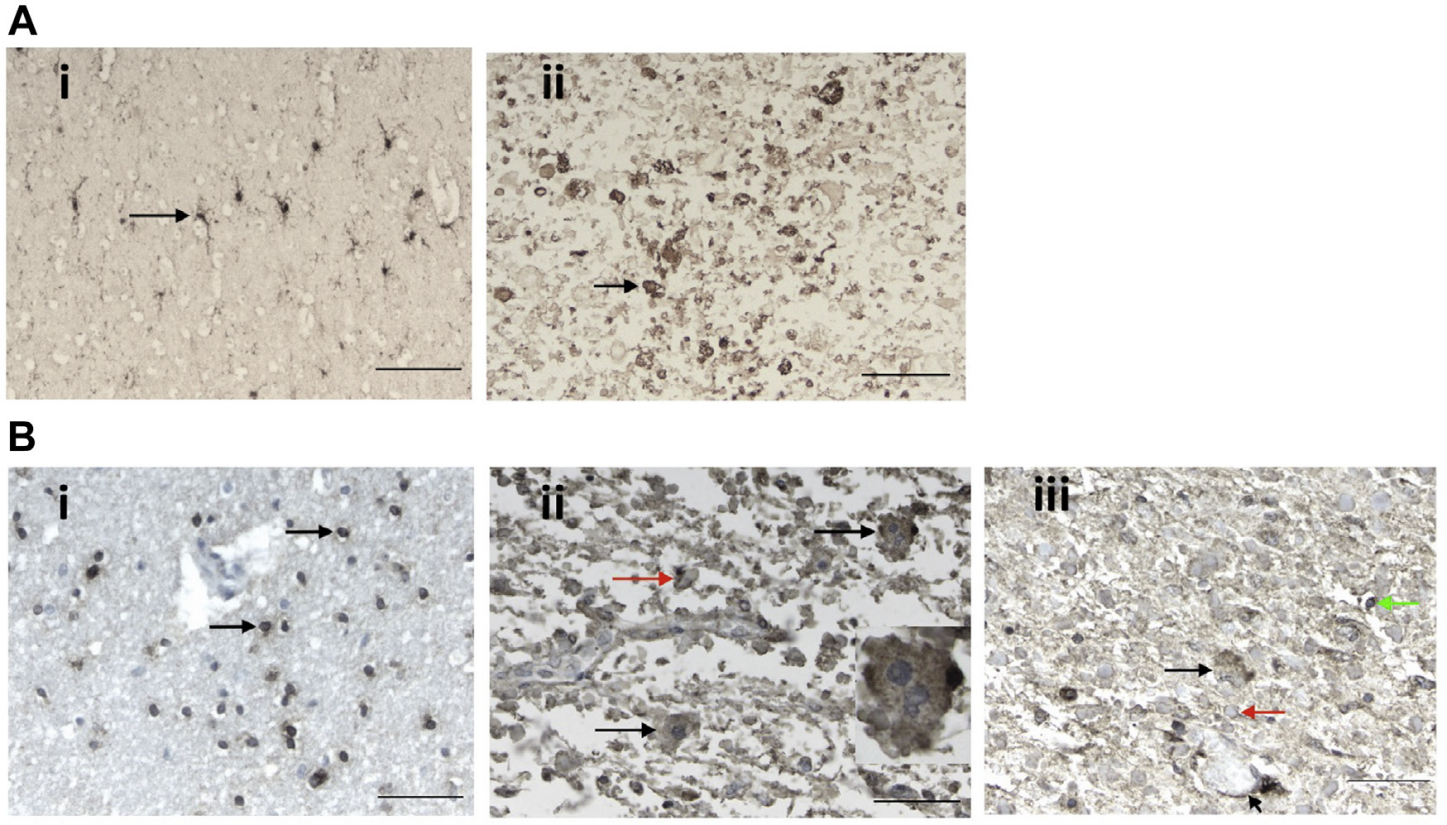
**AxD patient and human control immunohistochemistry analysis of FABP7 and UGT8.***A* and *B*, the demographics of the samples analyzed are as follows: AxD patient #1 is a 3-year-old female with an R239H GFAP mutation, and AxD patient #2 is an 8-year-old male with an R416W GFAP mutation, and the control is a 4-year-old female with no neuropathology. *A*, anti-FABP7 staining. *A*i, normal subcortical white matter from a control with no neuropathology. The antibody labels cells with several fine, branching processes, indicative of astrocytes. The *arrow* points to one astrocyte. The normal appearing white matter in the AxD patients is similar (data not shown). *A*ii, in this image of AxD white matter from AxD patient #1, the normal structures have been replaced by astrocytes and RFs, one of the largest is pointed out (*arrow*). The scale bars are 100 μm. *B*, anti-UGT8 staining. *B*i, normal subcortical white matter from a control with no neuropathology. The antibody labels the cytoplasm of small cells with round nuclei, indicative of oligodendrocytes (*arrows* point to two oligodendrocytes). Just above the *bottom* oligodendrocyte is a blood vessel, which is negative. *B*ii, in this image of subpial area of the cerebral cortex of patient #2, where RFs accumulate, astrocytes contain punctate, positive signal in the cell body (*black arrows*). *Round* to *oval* profiles represent RFs (*red arrow*), many of which stain peripherally, a common feature of RF immunostaining. The astrocyte at the *top right* is multinucleated, a common feature of AxD astrocytes and is shown at higher magnification in the *inset*. *B*iii, in this section of AxD subcortical white matter from AxD patient #1, showing an astrocyte (*black arrow*), positive staining of astrocyte end feet around a blood vessel (*short arrow*), RFs (one shown with *red arrow*), and an oligodendrocyte (*green arrow*). In the normal appearing white matter in the AxD sections, the antibody stained only oligodendrocytes, as in controls (data not shown). Scale bars are 50 μm.

To next determine whether FABP7 and UGT8 had unique staining patterns in the brain tissue that differed from *GFAP*^*Tg*^;*Gfap*^+/*R236H*^ mice, immunohistochemistry was performed (Fig. 7). The elevation in FABP7 was observed in astrocytes from an AxD patient *versus* control. However, the results for UGT8 immunohistochemistry did not clearly indicate a depletion of UGT8 in oligodendrocytes, and moreover in the subpial regions the UGT8 antibody reacted with RFs, which was not observed in the *GFAP*^*Tg*^;*Gfap*^+/*R236H*^ mice.

## Discussion

The *GFAP*^*Tg*^;*Gfap*^+/*R236H*^ mouse model of AxD provides a means to assess the contribution of astrocytes to a neurological disorder, and in recent years there has been a growing body of evidence indicating that astrocytes play a critical role in the pathological processes occurring in other neurological conditions including autism (8), epilepsy (9), ALS (10, 70), MDD (11), TBI (12), Huntington disease (70), and multiple sclerosis (65). For example, astrocytes exert non-cell autonomous effects in multiple sclerosis by secreting factors that interfere with oligodendrocyte maturation (65). To date, this study represents the only application of high-resolution mass-spectrometry-based proteomics to interrogate the total brain proteome of an AxD model. New knowledge gained using the *GFAP*^*Tg*^;*Gfap*^+/*R236H*^ mice includes additional evidence for aberrant glutamate metabolism, for delayed astrocyte maturation, and upregulation of the PPAR signaling pathway, including Fabp7, a key element of gliosis in brain stab injury models (40). Furthermore, previously uncharacterized effects were found on other brain cell types including a drastic downregulation of the oligodendrocyte-specific Ugt8 protein.

A primary function of astrocytes is the regulation of glutamate *via* the Slc1a2 transporter following excitatory neurotransmission (54). Consistent with earlier reports (15, 16, 18, 23), decreases were observed in this transporter in the *GFAP*^*Tg*^;*Gfap*^+/*R236H*^ mice relative to wild type. This alteration supports the conclusion that *GFAP*^*Tg*^;*Gfap*^+/*R236H*^ mice are defective in glutamate uptake, which may contribute to seizures that occur in some AxD patients and animal models. The results in this study suggest two potential mechanisms contributing to glutamate dysregulation. First, significant reductions in *GFAP*^*Tg*^;*Gfap*^+/*R236H*^ mice in the carbonic anhydrase, Ca2, and the electrogenic sodium bicarbonate cotransporter, Slc4a4, may cause astrocyte acidification and the toxic release of glutamate, as reported previously in ischemic injuries (58). Second, defects in glutamate buffering in astrocytes have been demonstrated from increased levels of fatty acids such as LPA (71). This mechanism may operate in AxD, as we found that a protein responsible for the hydrolysis of LPA, Plpp3, was significantly decreased in the *GFAP*^*Tg*^;*Gfap*^+/*R236H*^ mice. There were also multiple astrocytic proteins altered in the *GFAP*^*Tg*^;*Gfap*^+/*R236H*^ mice related to fatty acid metabolism (Slc27a1, Fabp7, Acot1, Acsl6, Fasn), coupled with upregulation of the PPAR signaling pathway, which can be activated by fatty acids (42). These aberrant levels of fatty acids could explain the glutamate dysregulation that is pertinent to the phenotypes displayed by *GFAP*^*Tg*^;*Gfap*^+/*R236H*^ mice. For example, extracellular LPA inhibits glutamate uptake in astrocytes (71), direct injections of LPA into adult mouse striatum causes gliosis (72), and LPA causes demyelination when injected intrathecally into mice (73).

Various astrocyte cell surface and membrane proteins (Slc1a2, Gja1, Mlc1, Kcnj10, Slc38a3, S1pr1), which increase during maturation from nondiseases samples (63) (mouse P7 *versus* P32 astrocytes and fetal *versus* mature human astrocytes), were decreased in the *GFAP*^*Tg*^;*Gfap*^+/*R236H*^ mice brain tissues relative to the wild-type controls at P25 in this study. These decreased proteins relative to wild-type mice related to cell maturation proteins have critical functions in astrocyte biology, ranging from glutamate buffering (Slc1a2), establishing gap junction connexons to neighboring cells (Gja1), regulating the blood–brain barrier (BBB) development in perivascular astrocyte end feet (Mlc1) (74), potassium buffering (Kcnj10), regulating the efflux of glutamine (Slc38a3) (55), and the G-protein-coupled receptor for lysosphingolipid sphingosine 1-phospate (S1pr1), which is a molecular target for drugs that inhibit demyelination (75). The reduced levels observed for S1pr1 in *GFAP*^*Tg*^;*Gfap*^+/*R236H*^ mice may serve a protective function by attenuating demyelination in the model and explain why only subtle effects on myelination were detectable by RNA-seq and proteomics, but not by gross inspection of the CNS during autopsy (18).

Comparison of the results in this study to a database of proteins expressed predominantly from specific CNS cell-types reveals that numerous proteins with oligodendrocyte expression were decreased, albeit with the limitation that the database employed for this analysis was constructed from normal mouse brain rather than from diseased tissues (49). Depleted levels were detected in the six major protein constituents of myelin (Cnp, Mbp, Mog, Mobp, Mag, Plp1), a result previously observed at the RNA level in hippocampus tissue from *GFAP*^*Tg*^;*Gfap*^+/*R236H*^ mice (19). However, this prior transcriptomic study did not identify a change in Ugt8 expression as a possible upstream effector responsible for the observed deficits in these myelin components, perhaps because the RNA levels for Ugt8 were less severely decreased (*e.g.*, −1.7 fold) than the protein level. Ugt8 was also validated in this study because mice with deficits in ether lipids have subtle abnormalities in myelination (76), and *Ugt8* knockout mice recapitulate many phenotypic characteristics shared with *GFAP*^*Tg*^;*Gfap*^+/*R236H*^ mice ranging from tremors, mild ataxia, and hindlimb mobility impairment (66). By untargeted μDIA proteomics, PRM-MS, and Western blotting, Ugt8 was found to be significantly decreased by −4.3 fold, −3.7 fold, and −13.7 fold, respectively. Although the values for Western blotting *versus* the two mass-spectrometry-based methods did not yield the same fold-change results, each indicated a major reduction in Ugt8 protein beyond the small reduction found previously in the *Ugt8* RNA levels in the hippocampus of *GFAP*^*Tg*^;*Gfap*^+/*R236H*^ mice (19). The drastic reduction in Ugt8 protein compared with the modest reduction in oligodendrocyte-associated myelin protein components (Cnp, Mbp, Mog, Mobp, Mag, Plp1) suggests that Ugt8 was decreased by a more specific mechanism in *GFAP*^*Tg*^;*Gfap*^+/*R236H*^ mice than by a vast reduction in oligodendrocytes. Immunohistochemistry analysis indicated that deficits in Ugt8 were widespread in myelin tracts across all brain regions in *GFAP*^*Tg*^;*Gfap*^+/*R236H*^ mice, rather than being localized to a specific region subjected to severe gliosis, such as the hippocampus. Last, in Western blots from human AxD patients and controls, the amount of UGT8 appeared depleted but did not reach statistical significance, perhaps due to low sample number of this rare disease (<1 in 1,000,000 individuals). It is notable that upon analysis by immunohistochemistry, the staining in subpial brain regions in an AxD patient was particularly reactive to UGT8 suggesting an accumulation in RFs. However, UGT8 was not found previously in a proteomic study as a component of RFs from AxD patients (22).

Other glial cell types such as microglia are dysregulated at the mRNA level in *GFAP*^*Tg*^;*Gfap*^+/*R236H*^ mice (19). Specifically, in this report the proteome analysis found that the microglia-associated protein, Ctsd, and complement proteins (C1qa, C1qb, C1qc) were increased. These secreted microglia-associated proteins represent a particularly noteworthy finding given their ability to induce reactive astrocytes that exhibit a neurotoxic phenotype (68, 69).

Fatty acid binding proteins (Fabps) are critical regulators of lipid metabolism, energy homeostasis, and inflammation (77). The untargeted μDIA proteomics analysis found elevated levels of the brain-specific Fabp7 isoform in *GFAP*^*Tg*^;*Gfap*^+/*R236H*^ mice. Subsequently, Fabp7 was selected for additional analysis by PRM-MS and immunohistochemistry because deletion of the Fabp7 gene in mice results in reduced proliferation of reactive astrocytes following cortical stab injuries (40), which shares some clinical features to AxD including a reactive gliosis phenotype with upregulation of Gfap. An additional rationale for investigating Fabp7 was based on a recent report demonstrating that Fabp7 is upregulated in a mutant human superoxide dismutase 1 (hSOD1) mouse model of ALS and causes an NF-κB-driven inflammatory response that is damaging for motor neuron survival (78). In the context of AxD, NF-κB may serve to increase Gfap to toxic levels, since NF-κB (Rela) was significantly upregulated in the untargeted proteomics dataset by 2.1-fold in *GFAP*^*Tg*^;*Gfap*^+/*R236H*^ mice, and its blockade by aspirin has been shown to lower Gfap levels in astrocytes (79). The PRM-MS results for Fabp7 confirmed its elevation in *GFAP*^*Tg*^;*Gfap*^+/*R236H*^ relative to wild-type mice of 1.9-fold, and Western blotting the levels of FABP7 in human AxD patient *versus* control samples indicated a fourfold significant increase. Immunohistochemistry analysis further verified the upregulation of Fabp7 in both the mouse model analyzed in this study and in an AxD patient *versus* human control. Consistent with the role of Fabp7 in reactive gliosis, the expression of Fabp7 and the number of Gfap+ cells suggest that the proliferation of reactive astrocytes was increased in *GFAP*^*Tg*^;*Gfap*^+/*R236H*^ mice, particularly in the hippocampus where acute reactive gliosis occurs.

Upregulation of Fabp7 in *GFAP*^*Tg*^;*Gfap*^+/*R236H*^ mice is anticipated to decrease the functional activity of the PPAR pathway (80), at least in part because Fabp7 and PPARs bind to many of the same ligands, including long-chain fatty acids (81). The upregulation of PPARα may serve as a protective mechanism in *GFAP*^*Tg*^;*Gfap*^+/*R236H*^ mice, since administering pharmacological agonists blocks neurodegeneration and neuroinflammation in murine models of ALS (82). Among the various PPAR isoforms, the strongest relationship observed in the untargeted μDIA proteomic dataset implicates the activation of PPARα in *GFAP*^*Tg*^;*Gfap*^+/*R236H*^ mice due to the heightened amounts of its corresponding transcriptional target proteins (Acot1, Acsl3, Hmgcs2, Acox1) (43, 44, 45), although it should be noted that the prior studies used to implicate the expression of these particular PPAR target genes were generated from nonbrain tissues and some downstream targets of the pathway are expressed by multiple PPAR isoforms (47, 48). Altogether, the upregulation of Fabp7 in *GFAP*^*Tg*^;*Gfap*^+/*R236H*^ mice and FABP7 in AxD patients is expected to induce a harmful phenotype by generating an NF-κB inflammatory response (78) resulting in heightened levels of Gfap (79) and decreasing the anti-inflammatory effects of the PPAR pathway (80, 81).

In summary, *GFAP*^*Tg*^;*Gfap*^+/*R236H*^ mice have alterations in many proteins associated with astrocyte cell maturation and fatty acid metabolism. Notably, reductions in proteins associated with astrocyte maturation were observed that are located at the cell membrane and cell surface. Additionally, changes in fatty acid metabolism were observed that are relevant to the *GFAP*^*Tg*^;*Gfap*^+/*R236H*^ mice by having the ability to dysregulate glutamate buffering by astrocytes (71), cause demyelination (73), and upregulate the PPAR pathway (42). This study also revealed that *GFAP*^*Tg*^;*Gfap*^+/*R236H*^ mice exhibit changes in other cell types beyond astrocytes, including Ugt8 deficits in oligodendrocytes and elevated levels of microglia-associated complement proteins.

## Data Availability

All of the raw proteomics data files corresponding to the untargeted proteomics and PRM datasets are available at the ProteomeXchange Consortium (http://proteomecentral.proteomexchange.org/) *via* the PRIDE partner repository with the dataset identifier PXD021884. MS2 chromatograms in HTML format are also available here for all the proteins quantified in the untargeted proteomics dataset.

## Supplemental data

This article contains supplemental data.

## Conflict of interest

The authors have no conflicts of interest in the study.
