# Supplementary material for: Metabolic Enzyme Alterations and Astrocyte Dysfunction in a Murine Model of Alexander Disease With Severe Reactive Gliosis
Source: Mol Cell Proteomics. 2021 Nov 20;21(1):100180. doi: 10.1016/j.mcpro.2021.100180 (PMC8717607; doi:10.1016/j.mcpro.2021.100180)
Supplement: Supplemental Data [file mmc1.docx]

**Metabolic Enzyme Alterations and Astrocyte Dysfunction in a Murine Model of Alexander Disease with Severe Reactive Gliosis**

Michael R. Heaven, Anthony W. Herren, Daniel L. Flint, Natasha L. Pacheco, Jiangtao Li, Alice Tang, Fatima Khan, James E. Goldman, Brett S. Phinney, and Michelle L. Olsen

**Supplementary Tables and Figures Legends**

Supplemental Table 1: List of all proteins, peptides, and precursor ions targeted for acquisition in the PRM-MS experiment.

Supplemental Table 2: Relative abundance of 5,005 proteins quantified in the untargeted proteomics experiment as well as differentially abundant proteins with a P-value < 0.05, fold-change > 1.3, and KEGG pathway results.

Supplemental Table 3: PRM-MS targeted assay results for proteins validated from the untargeted proteomics study.

Supplemental Table 4: Demographics of the AxD and control human samples analyzed by Western blot.

Supplemental Figure 1: Annotated MS2 chromatograms for all the peptide and protein measurements from the PRM-MS acquisition list 1.

Supplemental Figure 2: Annotated MS2 chromatograms for all the peptide and protein measurements from the PRM-MS acquisition list 2.
