# Supplementary material for: Metabolic Enzyme Alterations and Astrocyte Dysfunction in a Murine Model of Alexander Disease With Severe Reactive Gliosis
Source: Mol Cell Proteomics. 2021 Nov 20;21(1):100180. doi: 10.1016/j.mcpro.2021.100180 (PMC8717607; doi:10.1016/j.mcpro.2021.100180)
Supplement: Supplemental Figure S2 [file mmc3.zip › Supplemental Figure 2.html]

Protalizer Analysis - PRM List 2 AxD (Group 1 samples) vs wild type (Group 2 samples)
 


Protalizer Analysis - PRM List 2 AxD (Group 1 samples) vs wild type (Group 2 samples)

---

|  |  |
| --- | --- |
| Protein: |  |
| Peptide: |  |
| File: |  |
